# Distribution patterns and driving mechanisms of ciliate communities from continental shelf to deep basin of the Northeastern South China Sea

**DOI:** 10.1093/plankt/fbaf020

**Published:** 2025-08-12

**Authors:** Shaowei Wen, Zijun Cheng, Gang Li, Kaizhi Li, Yehui Tan, Weiwei Liu

**Affiliations:** State Key Laboratory of Tropical Oceanography, Guangdong Provincial Key Laboratory of Applied Marine Biology, South China Sea Institute of Oceanology, Chinese Academy of Science, Guangzhou 510301, China; University of Chinese Academy of Sciences, Beijing 100049, China; State Key Laboratory of Tropical Oceanography, Guangdong Provincial Key Laboratory of Applied Marine Biology, South China Sea Institute of Oceanology, Chinese Academy of Science, Guangzhou 510301, China; University of Chinese Academy of Sciences, Beijing 100049, China; State Key Laboratory of Tropical Oceanography, Guangdong Provincial Key Laboratory of Applied Marine Biology, South China Sea Institute of Oceanology, Chinese Academy of Science, Guangzhou 510301, China; Daya Bay Marine Biology Research Station, South China Sea Institute of Oceanology, Chinese Academy of Sciences, Shenzhen 518121, China; State Key Laboratory of Tropical Oceanography, Guangdong Provincial Key Laboratory of Applied Marine Biology, South China Sea Institute of Oceanology, Chinese Academy of Science, Guangzhou 510301, China; State Key Laboratory of Tropical Oceanography, Guangdong Provincial Key Laboratory of Applied Marine Biology, South China Sea Institute of Oceanology, Chinese Academy of Science, Guangzhou 510301, China; University of Chinese Academy of Sciences, Beijing 100049, China; State Key Laboratory of Tropical Oceanography, Guangdong Provincial Key Laboratory of Applied Marine Biology, South China Sea Institute of Oceanology, Chinese Academy of Science, Guangzhou 510301, China

**Keywords:** protozoa, microzooplankton, diversity, community assembly, co-occurrence network

## Abstract

Ciliates are common components of microplankton and play crucial roles in the marine microbial food web, but little is known regarding how community assembly and species association vary from shelf to basin. A survey of ciliate diversity was conducted in the northeastern South China Sea during the winter of 2013, and the spatial distribution, driving mechanisms and network relationships of ciliate community were revealed. Our results showed that ciliate community compositions were similar between continental shelf and basin areas but significantly different between photic and aphotic waters. Higher ciliate abundances were found in shelf and photic waters than in basin and aphotic waters, primarily due to differences in light, nutrients and food resources. Across the entire sea area, environmental factors had a more significant influence on communities than spatial factors, suggesting that deterministic processes played a significant role in ciliate distribution. Co-occurrence network analysis revealed that ciliate networks were more stable in basin and photic waters than in shelf and aphotic waters, which was probably related to more complex relationships in the former two waters. Combined with the broader niche breadth, our study suggested that the ciliate community in basin and photic waters would show a stronger defense capability against environmental changes.

## INTRODUCTION

Ciliates, as essential components of the marine microbial food web, significantly contribute to energy flow and nutrient cycling in marine ecosystems (Lynn, 2010). Functioning primarily as secondary consumers, they feed on phytoplankton and bacteria, facilitating energy transfer to higher trophic levels (Pomeroy, 1974). Moreover, many ciliates exhibit mixotrophic capabilities, acquiring chloroplasts from prey phytoplankton to conduct photosynthesis, thus enhancing marine carbon storage.

As the largest ecosystem on the planet, the open ocean possesses extremely high plankton biodiversity. A great number of investigations on diversity and distribution of ciliates have been conducted in open ocean (Dolan *et al.,* 2007; Gómez, 2007; Doherty *et al.,* 2010; Grattepanche *et al.,* 2016; Zhang *et al.,* 2017; Santoferrara *et al.,* 2018; Yang *et al.,* 2020; Huang *et al.,* 2021; Wang *et al.,* 2024). Some studies revealed that ciliates communities are homogeneously distributed in open area. For example, from west to east Mediterranean, no evident change was found in the structure of the ciliate community with respect to aloricate size-classes, mixotroph size-classes and contribution of mixotrophs to total abundance (Pitta *et al.,* 2001). Along a 163 km transect off the coast of New England abundant ciliate members show high similarity from inshore to offshore (Grattepanche *et al.,* 2015). The Tara and Malaspina studies also suggested significant spatial differences of ciliate assemblages in global distribution (Gimmler *et al.,* 2016; Canals *et al.,* 2020). The open ocean features remarkable hydrodynamic connectivity. This effectively facilitates the mixing and exchanging of ciliates, thereby bringing about the homogeneity of ciliate assemblages. However, in some other studies, significant spatial variations in ciliates community have been observed. For example, high abundance and species richness fluctuations of tintinnid species was found from western to central Pacific Ocean (Gómez, 2007). Distinct spatial groups of ciliate communities were detected among Northwest, North and South of South Chin Sea in terms of taxonomy, motility and feeding habit (Liu *et al.,* 2021). The spatial patterns of ciliate community structure could be clearly discriminated in different water mass in both East China Sea and South China Sea (Sun *et al.,* 2020; Yang *et al.,* 2020). In these studies, the investigation sites were mostly located in separate areas and combined with environmental heterogeneity factors such as water mass, which may drive ecological niche effects and shape different communities. Continental shelf, slope and ocean basin are typical habitat regions in the open sea. To date, less is known about the distribution patterns of ciliate community in the shelf-slope-basin continuum. Due to the spatial connectedness, there is an exclusive water exchange among these regions. Meanwhile, controlled by different physical processes, each region also has unique environmental characteristics (Sun *et al.,* 2021). Whether the ciliate assemblages are homogeneity or heterogeneity among these regions is still unknown.

Understanding the determining mechanisms of communities is a central goal of ecology (Zhou and Ning, 2017). Numerous studies have established a strong relationship between ciliate communities and environmental factors such as temperature, salinity, nitrates, phosphates and phytoplankton significantly influence ciliate growth and distribution (Liu *et al.,* 2024). On the other hand, stochastic dynamics deduced by spatial differentiation also contribute to the distribution of ciliate communities, and that dispersal limitation due to spatial distance is a primary driver of ciliate community variation (Sun *et al.,* 2020). Apart from environmental selection and dispersal effect, interspecific relationships also crucially affect the ciliate community assembling. Random processes of births-deaths and environmental filtering play important roles in regulating species–species interactions, which then control the community composition and stability (Sun *et al.,* 2021). Previous studies have examined the relative importance of environment and space on ciliate communities as well as the species interactions in a variety of aquatic environments such as coast area, sea ice, intertidal zone, deep seamounts (Sun *et al.,* 2020; Xu *et al.,* 2022; Yu *et al.,* 2022; Zhao *et al.,* 2023), but there is a knowledge gap in the determining mechanisms of ciliate in the shelf-slope-basin continuum. The shelf-basin area hosts both strong environment gradients and broad spatial scopes, which raise the question: In the shelf and basin regions, environmental selection or dispersal effect, which is the dominant driving factor for ciliate assemblage? Additionally, what are the interspecific relationships and community stability within each of these regions?

The northeastern South China Sea (NESCS), situated in the western Pacific, covered a broad area about 700 000 km^2^ of which the continental shelf and ocean basin each nearly take up half, while the proportion of slope is extremely small due to the sharply decreasing depth from shelf to basin. Therefore, significant spatial connectivity and distance gradients were exhibited in the shelf-basin continuum of NESCS (Su, 2004; Dai *et al.,* 2009). Meanwhile, the hydrographic characteristics of NESCS exhibit complex variations from shelf to basin (Hu *et al.,* 2000). Horizontally, shelf areas receive terrestrial inputs such as Pearl River discharge, resulting in nutrient-rich, low-salinity waters, while basin areas usually trap the water masses like Kuroshio intrusion and thus are characterized by nutrient-poor, high-salinity waters (Ding *et al.,* 2022). Vertically, the depth of NESCS shelf ranges from 20 m to 200 m, while deep basin has a mean depth 1 200 m with thermocline depth around 30 m. Furthermore, the mixing of water layers were commonly found in shelf area due to the effect of coastal upwelling, while in basin area, significant environmental difference prevail between epipelagic and mesopelagic zones especially in terms of light, temperature, salinity and nutrients (Cai *et al.,* 2020). Overall, the strong environmental heterogeneity, along with the broad spatial range, makes NESCS an ideal area for studying biogeography.

Here, we investigated the distribution patterns of ciliate communities in the shelf-basin waters in NESCS and assessed the impact of spatial and environmental factors on community assembly. This study aims to answer these questions: (i) The connectivity of open ocean contributed to exclusive water exchange from shelf to basin, while each region has unique environmental characteristics effected by different physical processes. Whether the ciliates form homogeneous or heterogeneous assemblages in regional space? (ii) The shelf-basin area host both strong environment gradients and broad spatial scope in vertical and horizontal directions. Environmental selection or dispersal effect, which exerts a more significant influence on the structure of ciliate communities? Whether the interspecific relationships and community stability variated among different spatial regions? Our findings will provide novel insights into the ecological mechanisms of microzooplankton community assembly in tropical oceanic province.

## MATERIALS AND METHOD

### Study area and sampling stations

The survey was conducted by the research vessel “Shiyan 3” from November 6 to December 5, 2013. A total of 34 stations were investigated in the NESCS (19°00′-23°18’N, 115°00′-120°30’E), covering the continental shelf, slope and basin areas, with bottom depths ranging from 21 to 4 108 m (Fig. S1). Water samples were collected at depths of 0 m, 25 m, 50 m, 75 m, 100 m, 200 m, 300 m, 500 m, 1 000 m and 1 500 m using Niskin bottles mounted on a CTD rosette (Sea-Bird 911 plus).

### Environmental parameters

Standard methods were used to measure environmental variables, including salinity (Sal), temperature (Tem), pH, dissolved oxygen (DO), abundance of microzooplankton Synechococcus (Syn), Picoeukaryotes (Peuk) and Prochlorococcus (Pro), as well as size-fractionated chlorophyll a concentration, including microplankton chlorophyll a (MChla, > 20 μm), nanoplankton chlorophyll a (NChla, 3–20 μm) and picoplankton chlorophyll a (PChla, < 3 μm). Detailed methods have been described in previous studies (Li *et al.,* 2017).

### Ciliate identification and analysis

Water samples (2 L) were fixed with Lugol’s solution and stored in the dark until analysis. In lab, samples were gradually concentrated to 50 mL. Subsamples (1 to 5 mL) were taken into a counting chamber, and all ciliates in the chamber were identified and counted under an inverted microscope at magnifications of 200x. All ciliates were identified to the species level following (Hu *et al.,* 2019; Kofoid and Campbell, 1929). Uncertain individuals were picked out and identified using protargol impregnation (Wilbert, 1975).

Additionally, the ecological trait types of ciliates, including motility and feeding habits, were elucidated. Species were classified into three motility types, i.e. planktonic, sessile or vagile, based on literature (Coppellotti and Matarazzo, 2000; Xu *et al.,* 2009).For feeding habits, species were categorized into five types: detritivores, bacterivores, carnivores, predators and non-selective feeders, based on original species descriptions and broader literature (Pratt and Cairns, 1985; Fernandez-Leborans, 2001; Fernandez-Leborans and Fernandez-Fernandez, 2002; Lynn, 2010).

### Data analysis

The spatial distribution of ciliate communities were analyzed based on two horizontal groupings, i.e. continental shelf (bottom depth of sample site was less than 200 m, containing 59 samples) and slope-basin area (bottom depth of sample site was more than 200 m, slope and basin areas were combined because the proportion of slope is extremely small in NESCS (Su, 2004), containing 112 samples) and two vertical groupings, i.e. photic zone (water depth was less than 200 m, containing 136 samples), and aphotic zones (water depth was more than or equal to 200 m, containing 35 samples). Considering that the basin area includes deep—water samples while shelf area does not, the ciliate communities of continental shelf and basin area were also compared based on the samples from the photic zone. Analysis of similarity (ANOSIM) and non-metric multidimensional scaling ordination (NMDS) analyses were used to analyze and visualize community differences among different groups. Indicator species analysis was performed to identify the species that characterized each area using the package “Indicspecies” in R (Dufrêne and Legendre, 1997). Only species with indicator values (IV) > 0.3 and *P* < 0.05 were considered good indicators.

Pearson correlation was used to analyze the relationship between ciliate abundance/species α diversity parameters (Shannon diversity index and species richness SR) and environmental factors. To quantify the relative contribution of environmental and spatial factors in ciliate community assembly, variation partitioning analysis (VPA) with adjusted R^2^ coefficients based on redundancy analysis or canonical correspondence analysis (CCA) and Mantel test analysis were used. Spatial variables were generated using PCNM analysis based on the longitude and latitude of sampling sites. Forward selection was conducted to select significant explanatory variables (*P* < 0.05) for further analyses. All analyses were performed using the “vegan” package in R (R Core Team, 2021). Four co-occurrence networks were constructed based on ciliate communities from the four spatial groupings using the Spearman rank correlation coefficient (r) between species, with the “Hmisc” and “graph” packages in R. Only robust (Spearman |r| > 0.01) and statistically significant (BH-adjusted *P* < 0.05) correlations were considered, and visualization was performed using Gephi 0.10.1. Topological properties including edge number, node number, MD (modularity), CC (average clustering coefficient), APL (average shortest path length), GD (graph density), ND (network diameter) and AD (average degree), were calculated for each network as mentioned in a previous study (Telesford *et al.,* 2011). More nodes and edges, and high clustering coefficient, average degree, and graph density mean more complex structure and strong connection, which are essential for maintaining the balance and stability of the ecosystem. While excessive modularity, high average path length and network diameter suggest simple and sparse interactions among species, which tend to be less resilient to disturbances (Zhu *et al.,* 2021). Network stability was also assessed based on robustness test by simulating a network attack scenario in which nodes were gradually removed randomly (Peng and Wu, 2016). When nodes in the static network were removed, the loss of natural connectivity was measured. The higher natural connectivity indicates higher stability of community. To evaluate the network flexibility for environmental disturbance, niche breadth was calculated according to Pandit *et al.* (2009), using Levins’ niche breadth index (Pandit *et al.,* 2009). An organism group with wider niche breadth can be expected to be more flexible to adapt environment change.

## RESULTS

### Environmental characteristics

Principal Component Analysis (PCA) of environmental data showed an overlap of samples from the continental shelf and basin areas, but these in photic and aphotic waters were clearly separated (Fig. S2), which indicate that the environmental differences are not significant between the horizontal groupings but significant between vertical groupings. Additionally, the sample distribution in the aphotic water was relatively concentrated, while that in the photic water was more dispersed, indicating the higher consistency of environmental conditions among samples in aphotic than photic waters.

### Ciliate community composition and distribution

A total of 171 samples from 34 stations were analyzed, and 265 species belonging to 11 class/subclass taxa were detected. The ciliate communities were dominated by the subclasses Oligotrichia (relative abundance 43.6%) and Choreotrichia (32.8%), followed by classes Prostomatea (15.1%), Oligohymenophorea (4.1%) and Litostomatea (3.1%).

**Fig. 1:**
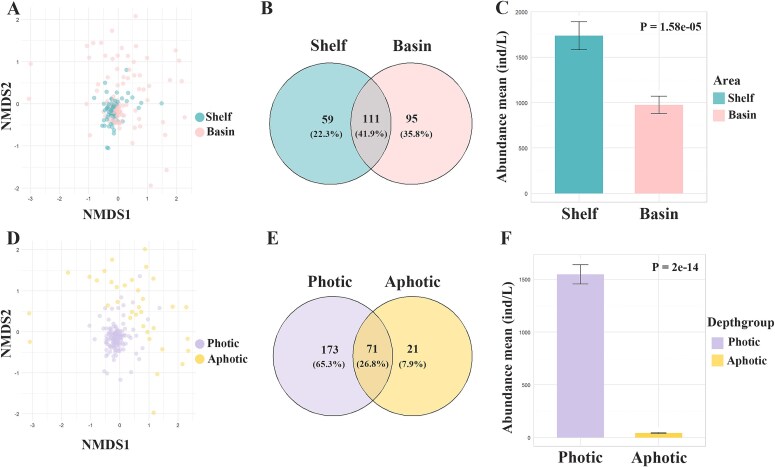
Comparison of ciliate communities in different spatial groups. NMDS analysis showing the distribution of ciliate samples in continental shelf and basin areas (**A**) and in photic and aphotic (**D**); Proportions of shared and endemic ciliate species in continental shelf and basin areas (**B**) and in photic and aphotic zones (**E**); Comparison of ciliate abundance in continental shelf and basin areas (**C**) and in photic and aphotic zones (**F**). Samples number: 59 in Shelf and 112 in Basin area, 136 in Photic and 35 in Aphotic zone.

**Fig. 2:**
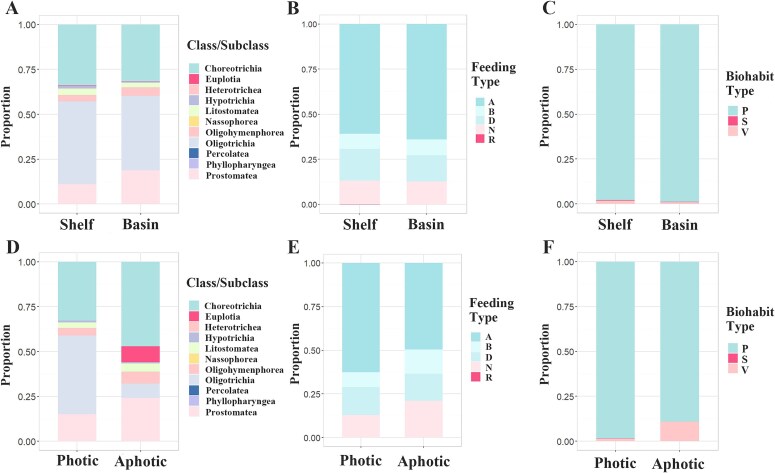
Comparison of ciliate species composition in different spatial groups. Taxonomic composition at class/subclass level to show their relative abundance in continental shelf and basin areas (**A**) and in photic and aphotic (**D**); Feeding type composition in continental shelf and basin areas (**B**) and in photic and aphotic (**E**); Biohabit type compositions in continental shelf and basin areas (**C**) and in photic and aphotic zones (**F**). A (Algivore), B (Bacterivore), D (Detritivore), N (Non-selective), R (Raptor), P (Planktonic), S (Sessile) and V (Vagile).

The ciliate communities in continental shelf and basin waters were compared. ANOSIM results (R = -0.018, *P* = 0.654) showed no significant difference between the two groups (Fig. S3A). NMDS results showed considerable overlap of samples from the two groups, indicating that the two communities could not be separated (Fig. 1A). In terms of species distribution, the number of endemic species was lower in the continental shelf than that the basin area (59 species vs. 95 species). The species shared between the two areas accounted for 41.9% of the total species (Fig. 1B), indicating a certain degree of ecological overlap and species exchange between the two areas. Additionally, the average abundance in continental shelf samples was higher than in basin samples (Fig. 1C). Regarding species composition, the dominant groups in both areas were the same, i.e. Choreotrichia, Oligotrichia and Prostomatea, although their relative abundances slightly varied(Fig. 2A). Similar results were found in feeding type and biohabit type compositions, algivorous and planktonic ciliates respectively dominated the community in both areas (Fig. 2B and C). In addition, indicator species were respectively identified for continental shelf and basin, which showed most indicator species belong to Oligotrichs and Choreotrichs in both areas (Fig. S4A). We also compared the communities of continental shelf and basin areas based on the samples from the photic zone. These showed similar results to the above analysis (Figs S5 and S6)and e.g. no significant differences were revealed between the two areas by ANOSIM and NMDS, and their species compositions were generally consistent. This indicated that the depth difference between the two areas did not affect their community structures.

When exploring the ciliate distribution in photic and aphotic waters, NMDS showed a clear separation of communities from the two groups (Fig. 1D), and ANOSIM analysis (R = 0.927, *P* = 0.001) provided statistical significance for the difference between them (Fig. S3B). In addition, the average abundance in the photic zone was much higher than in the aphotic zone (Fig. 1F). In terms of species richness, the number of species uniquely found in the photic water was much higher than that in the aphotic water (173 species vs. 21 species). The species shared between the two areas accounted for only 26.8% of the total species, indicating significant differences in species composition between the two areas (Fig. 1E). This was confirmed by our results of taxonomic composition comparison, which showed that the dominant groups were clearly different between the two zones, i.e. Oligotrichia dominated the photic water while Choreotrichia dominated the aphotic water. Additionally, the Euplotia and Prostomatea groups had higher proportions in the aphotic water compared to the photic water (Fig. 2D). In terms of feeding types, algivorous ciliates had a higher proportion in the photic water compared to the aphotic water (photic 62.55% vs. aphotic 49.41%), while bacterivorous (photic 8.57% vs. aphotic 14.12%) and non-selective feeders (photic 12.82% vs. aphotic 21.18%) had higher proportions in the aphotic than photic water. Predatory ciliates were only observed in the photic water layer (Fig. 2E). Biohabit type composition showed that planktonic ciliates dominated in both areas, but vagile ciliates were more prevalent in the aphotic than photic water (photic 1.33% vs. aphotic 10.65%) (Fig. 2F). In addition, indicator species analysis showed the aphotic water had significantly more indicator species than the photic water, and most indicator species in aphotic water belonged to the order Tintinnida (Fig. S4B).

### Relationship between ciliate communities and environmental factors

**Fig. 3:**
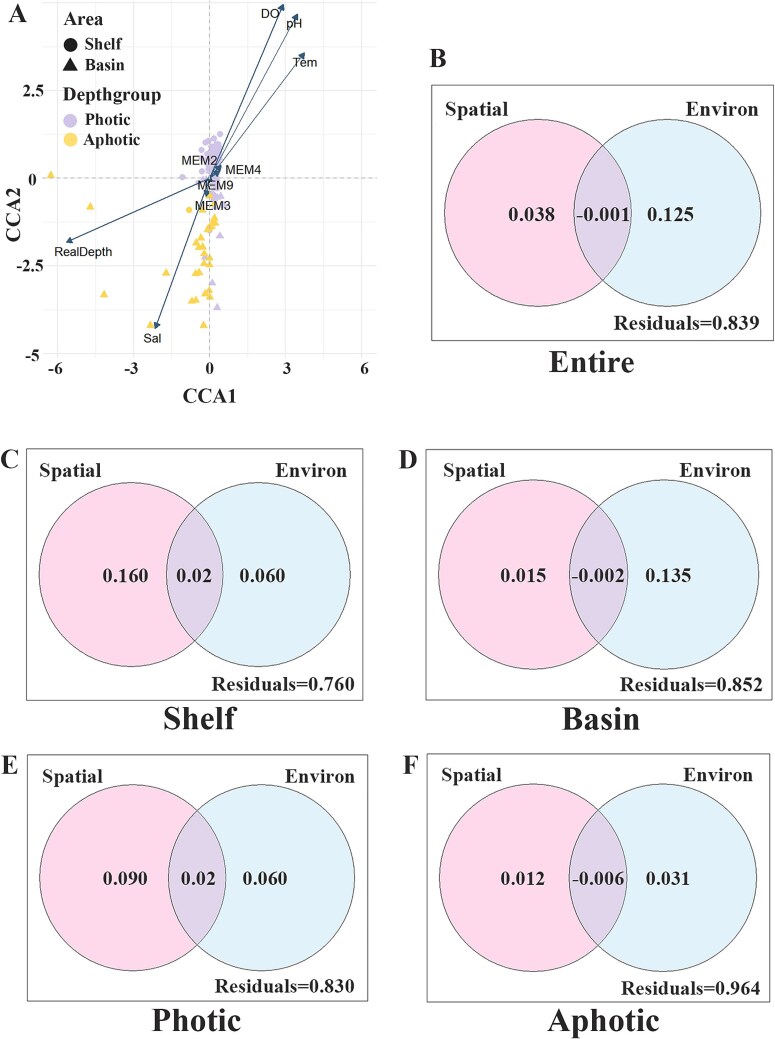
The driving factors of ciliate community. (**A**) CCA ordination showing the ciliate communities across the entire sea area in relation to the various factors; (**B**, **C**, **D**, **E**, **F**) VPA analysis revealing the contributions of environmental and spatial variables to ciliate community structure in the entire sea area (B), continental shelf (C), basin (D), photic zone (E) and aphotic zone (F), respectively.

For the entire sea area, Pearson correlation analysis showed that ciliate abundance was positively correlated with all biotic factors (Chla, Mchla, Nchla, Pchla, Syn, Pro and Peuk) and most physical factors (Tem, pH, DO), whereas it was negatively correlated with Sal. The Shannon index and species richness (SR) had low correlations with all environmental factors (Fig. S7). Regarding community composition, CCA analysis indicated that water depth, DO, pH, Sal and Tem were the main environmental factors affecting ciliate community (Fig. 3A). Additionally, the gradient changes of environmental factors (i.e. arrow directions) were in accord with the vertical variation of ciliate community (i.e. distributions in depth groups of community samples), indicating that environmental factors had a more significant impact on community variations between the depth groups. VPA analysis compared the contribution of spatial and environmental factors to community distribution (Fig. 3B), showing that environmental factors had a significantly higher independent explanatory power (12.5%) for community structure differences than spatial factors (3.8%). Mantel test results showed a significant positive correlation between environmental factors and ciliate communities (Table I. r = 0.6919, *P* = 0.001), while the correlation between spatial factors and communities was not significant (*P* = 0.193), further confirming that environmental factors had a more significant impact on ciliate community structure than spatial factors.

**Table I:** Mantel test for the correlation between ciliate community and spatial/environmental factors

	Factor	r	p
Entire	Spatial	0.0268	0.193
	Environ	0.6919	0.001
Shelf	Spatial	0.1436	0.023
	Environ	0.1359	0.049
Basin	Spatial	0.0276	0.236
	Environ	0.6748	0.001
Photic	Spatial	0.1196	0.012
	Environ	0.3559	0.001
Aphotic	Spatial	0.0655	0.174
	Environ	0.3537	0.005

The influence of environmental and spatial factors on ciliate community structure in different spatial groups was compared. VPA analysis results showed that in the continental shelf area, spatial factors had a higher explanatory power than environmental factors (Fig. 3C. 16% vs. 6%), whereas in the basin area, environmental factors had a higher explanatory power than spatial factors (Fig. 3D. 13.5% vs. 1.5%). Mantel test results further confirmed these findings (Table I): in the continental shelf area, both spatial and environmental factors showed significant positive correlations with community structure (*P* = 0.023; *P* = 0.049), but the r value for spatial factors was higher (0.1436 vs. 0.1359); whereas in the basin area, community structure was not significantly correlated with spatial factors (*P* = 0.236) but environmental factors (*P* = 0.001). Referring to the vertical groups, VPA showed that in the photic water, spatial factors had a higher explanatory power than environmental factors (Fig. 3E. 9% vs. 6%), whereas in the aphotic water, environmental factors had a higher explanatory power than spatial factors (Fig. 3F. 13.5% vs. 1.5%). Mantel test showed that both spatial and environmental factors had significant positive correlations with photic community structure (Table I. *P* = 0.012; *P* = 0.001), but the r value for spatial factors was higher (0.3559 vs. 0.1196); whereas in the aphotic water, environmental factors were significantly correlated with community structure (*P* = 0.005) but spatial factors were not (*P* = 0.174).

### Ciliate community co-occurrence network

**Fig. 4:**
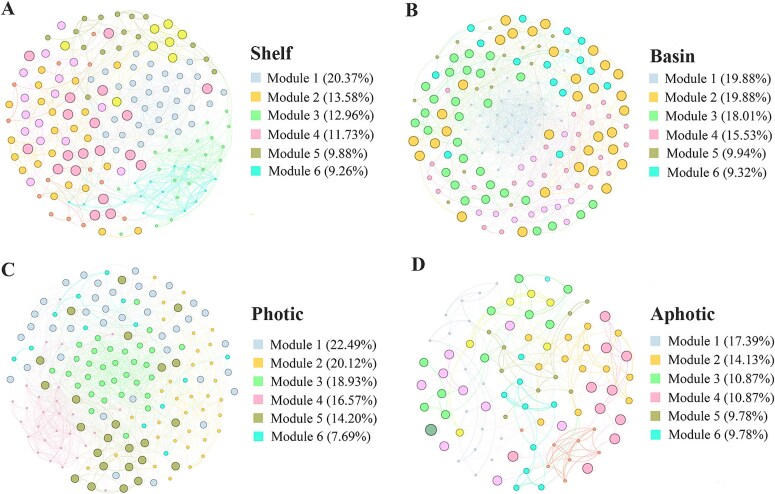
Co-occurrence networks of ciliate community species in different spatial groups. (**A**) networks in the continental shelf area; (**B**) networks in the basin area; (**C**) networks in the photic zone; (**D**) networks in the aphotic zone. Nodes are colored according to their modular groupings within the network.

**Fig. 5:**
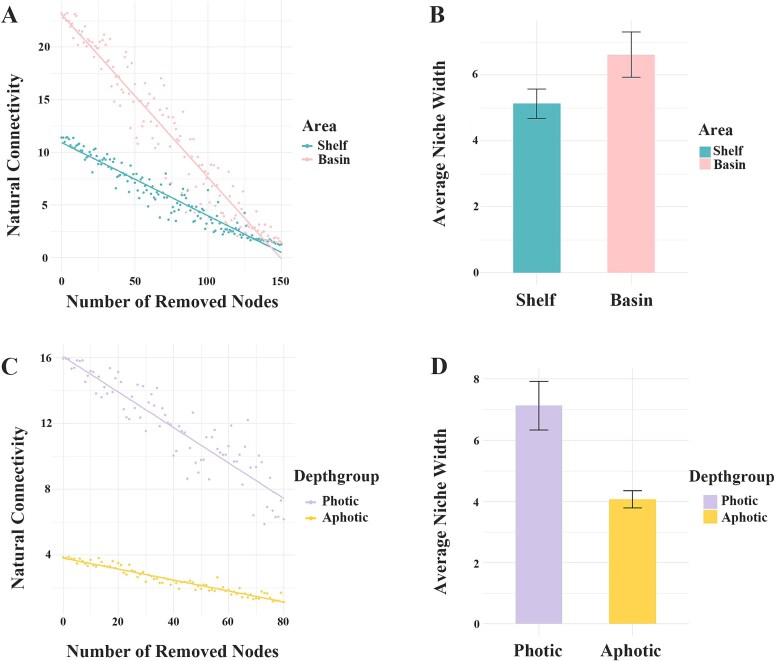
Comparisons of ciliate network stability and community niche breadth in different areas. (**A**, **C**) Robustness measured as the natural connectivity with the taxa randomly removed from communities in continental shelf and basin areas (A), photic and aphotic zones (C). The higher natural connectivity indicates higher stability of community; (**B**, **D**) Comparison of average niche breadth of ciliate species between the continental shelf and basinnetworks (B), and between photic and aphotic zones networks (D). The group with wider niche breadth were expected to be more flexible to adapt environment change.

Co-occurrence networks were constructed for ciliate communities in different areas (Fig. 4). Compared to the continental shelf community network, the basin community network had a similar number of nodes (162 vs. 161) but more edges (1747 vs. 2 397) (Table II). The basin network had a lower network modularity (MD) than the continental shelf network (0.444 vs. 0.554), indicating less niche differentiation in the basin environment. Additionally, the clustering coefficient (CC 0.476 vs. 0.409), average degree (AD 29.776 vs. 21.568) and graph density (GD 0.186 vs. 0.134) were higher but average path length (APL 0.624 vs 0.876) and network diameter (ND 1.337 vs. 1.942) were lower in the basin than continental shelf networks, suggesting closer connections among species in basin than continental shelf community. Network stability was assessed by robustness tests based on natural connectivity. The results showed that natural connectivity decreased with node removal in both continental shelf and basin networks, but the connectivity values were generally higher in the basin than continental shelf network throughout the node removal process (Fig. 5A), suggesting a stronger stability in the basin network. Furthermore, the niche breadth of ciliate species was calculated in the two areas, which showed that average niche breadth was wider in the basin community than continental shelf community (Fig. 5B), indicating the species in the basin area can utilize a wider range of resources than continental shelf area.

**Table II:** Comparison of co-occurrence network topological properties of ciliate communities indifferent groups (based on relative abundance > 0.01; r value > 0.01 and p-value < 0.05)

	N	E	MD	CC	APL	GD	ND	AD	r
Shelf	162	1747	0.554	0.409	0.876	0.134	1.942	21.568	6.679
Basin	161	2 397	0.444	0.476	0.624	0.186	1.337	29.776	7.461
Photic	169	2 252	0.483	0.367	0.513	0.159	0.988	26.651	8.364
Aphotic	92	512	0.723	0.468	1.751	0.122	3.144	11.130	4.601

N, number of nodes; E, number of edges; MD, modularity; CC, clustering coefficient; APL, average path length; GD, graph density; ND, network diameter; AD, average degree; r, small-world coefficient r = (CC/CC random)/(APL/APL random), where r > 1 indicates “small-world” properties, i.e. high interconnectedness and high efficiency (Telesford *et al.,* 2011).

Regarding the vertical groups, the photic water community network had more nodes (169 vs. 92) and more edges (2 252 vs. 512) than the aphotic water community network (Table II). Additionally, the average path length (APL 1.751 vs. 0.513), network diameter (ND 3.144 vs. 0.988) and modularity (MD 0.723 vs. 0.483) were higher but average degree (AD 11.130 vs. 26.651) and graph density (0.122 vs. 0.988) were lower in the aphotic water network, which indicated a looser network structure in aphotic water community. Robustness tests for network stability showed that natural connectivity decreased with node removal in both areas but connectivity values were consistently higher in the photic than aphotic water throughout the node removal process (Fig. 5C), which suggested a stronger stability in the photic water network. Furthermore, the average niche breadth of ciliate species was wider in the photic water community than the aphotic water (Fig. 5D).

## DISCUSSION

### Ciliate community shows high similarity between shelf and basin areas but clear difference between photic and aphotic zones in NESCS

Our alpha and beta diversity results indicate the differences of ciliate communities between continental shelf and basin areas are not significant (Fig. 1A and B; 2A–C). The results were inconsistent with some other studies on the distribution of ciliates in open ocean, in which significant community variations were found along cross-shore direction. For example, in Ross Sea, Antarctica, the ciliate community structure significantly variated between nearshore and offshore, and their diversity indices decreased from nearshore to offshore (Yu *et al.,* 2022). Off the coast of New England, USA, both morphospecies and OTUs data showed some tintinnid species found inshore were completely undetectable offshore and vice versa (Santoferrara *et al.,* 2016). Environmental heterogeneity was considered as the main contributor for the spatial variations in above studies. In our study, however, the environment characteristics were not significantly different between shelf and basin, as revealed by our PCA that environmental samples were generally overlapped due to their high similarity. The shelf and basin areas were closely located in the NESCS and have extensive hydrodynamic connectivity with each other (Xue *et al.,* 2004; Nan *et al.,* 2015), which promotes the exchange of water between the two areas, resulting in the weak difference in environmental conditions and the ciliate communities.

In the vertical direction, significant differences of ciliate communities have been observed between photic and aphotic water zones in our results. The environmental conditions such as temperature, light and DO were significantly variated in the two depth zones, which contributed important impacts on planktonic community (Wu *et al.,* 2018; Li *et al.,* 2021). Our results indeed showed a clear difference in the environmental data between photic and aphotic water zones (Fig. S3), which thus can explain the difference of ciliate communities. On the other hand, the physiological characteristics of ciliate also played a key role in their vertical distribution. The unique ecological habit determined their niches which limited them to specific water depth. For example, our community composition analyses showed that the Oligotrichia group has the highest proportion in the photic water, while the Choreotrichia group dominates in the aphotic water. The Oligotrich ciliates are primarily mixotrophic or predators of phytoplankton, and thus more adapt to the photic water where the light is sufficient for their photosynthesis and their prey. The Choreotrichs mainly consist of tintinnids which possess a lorica attaching their bodies (Lynn, 2010). Studies have shown that the lorica of tintinnid ciliates adds extra weight, which thus increases the possibility of sinking (Suzuki and Taniguchi, 1995) and makes them more likely to be observed in the deep water (Fernandes, 2004). The dominance of tintinnids in deep water was also confirmed by our indicator species analysis which revealed that all indicator species of deep water belong to tintinnid ciliates. Regarding feeding types, the proportion of algivorous ciliates was higher in the photic than aphotic water, while that of bacterivorous ciliates increased from photic to aphotic water. Considering that the concentrations of phytoplankton and bacteria are higher in the photic and aphotic water layers, respectively (Herndl *et al.,* 1997; Mellard *et al.,* 2012; Kuuppo-Leinikki and Salonen, 1992), it is understandable that their predators, algivorous and bacterivorous ciliates, have higher abundances in their dominated water depth. In terms of biohabits, vagile ciliates are more prevalent in the aphotic than photic water. Vagile ciliates usually like to move independently on the surface of substrates with their cilia (Xu *et al.,* 2009). The presence of a large amount of settling detritus in the aphotic sea might provide sufficient attachment substrates for vagile ciliates.

With regard to abundance, ciliates exhibited higher values in the shelf and photic waters compared to the basin and aphotic waters. Similar results have been found in other studies (Santoferrara and Alder, 2009; Gong *et al.,* 2020; Huang *et al.,* 2021; Liu *et al.,* 2021; Yu *et al.,* 2022). Ciliate abundance is closely related to food availability (Sherr and Sherr 2002). Previous studies in the South Pacific Ocean have revealed that Chla is the key impacting factor of ciliates abundance as an indicator of food resource (Dolan *et al.,* 2007, 2016). Agreed with this, our Pearson correlation analysis also showed that ciliate abundance is significantly positively correlated with food factors such as Chla, Syn, Peuk and Pro. In the shelf area, the terrestrial input of nutrients is high, which enriches the food sources for ciliates and promotes their growth. In the photic water, the light and temperature are more suitable for phytoplankton growth, which thus also provide sufficient food source for ciliate and support their high abundance.

### The influence of spatial and environmental factors on ciliate community assembly varied in different areas

Our CCA results showed that both spatial and environmental factors played important roles in the distribution of ciliates in the NESCS. The relationship between environmental factors and biological communities reflects the influence of environmental selection in community, indicating that deterministic processes play roles in community construction. In contrast, the influence of spatial factors on biological communities suggest the driving role of dispersal limitation in community distribution, indicating that stochastic processes contribute to community assembly(Niu *et al.,* 2009; Yang *et al.,* 2023). Our VPA and Mantel tests revealed that environmental factors had more explanation on community distribution in the entire NESCS area than spatial factors did, suggesting that deterministic processes contribute more to the distribution of ciliates than stochastic processes. This is inconsistent with the findings of a previous study in the South Pacific (Dolan *et al.,* 2007), where the distribution of tintinnid ciliates was found to match the neutral theory model, suggesting that stochastic processes have a greater impact on the community. This difference might be related to locations of research areas. NESCS is a marginal sea which is impacted by various regional hydrodynamic processes such as Pearl River discharge and coastal upwelling in coastal area, Kuroshio intrusion in eastern open are (Ding *et al.,* 2022). As a result, the NESCS exhibits significant environmental heterogeneity and high diversity of ecological patches, which thereby explains the notable influence of deterministic processes on community distribution. In contrast, the research area in Dolan *et al.* (2007) covered nearly half Pacific Ocean, where environmental characteristics were generally homogeneous and planktonic community mostly distributed by random dispersal, and thus stochastic processes played more roles in community assembly.

The influences of spatial and environmental factors on ciliate community assembly in regional areas of NESCS were also compared, which showed variated results. In the shelf area, spatial factors have a more significant impact in community than environmental factors, while in the basin area, environmental factors become the main drivers of community assembly. The shelf area is extensively influenced by the water masses of China coastal currents during our investigation. Strong physical processes promote the dispersal of microplankton, leading to a significant impact of stochastic processes on community distribution (Sun *et al.,* 2021). On the other hand, the high terrestrial input of nutrients provides sufficient food sources for ciliates in shelf area, which reduces the competitive pressures built by environmental selection and thus results in the weak impact of deterministic processes (Dini-Andreote *et al.,* 2015). In contrast, the basin area harbors deep-sea habitats which increase the environmental heterogeneity and niche diversity, thereby explaining the notable influence of deterministic processes on community distribution. For vertical groupings, our results showed that spatial factors slightly outperformed environmental factors in explaining ciliate community distribution in the photic water layer. As mentioned above, there are vital hydrodynamic processes such as the current, eddy and internal wave in the NESCS. These processes mainly happen in the photic water and significantly promote the dispersal of microplanktons(Ding *et al.,* 2022). Therefore, the high contribution of stochastic processes to ciliate community assembly is easy to understand. Little was known about the assembly process of microbial communities below the photic zone. Our study revealed that in the aphotic water, environmental factors have a slightly greater impact on community than spatial factors. Previous study showed that the influence of environmental factors on protists becomes more significant relative to spatial factors with increasing depth (Wu *et al.,* 2018). In deep water like aphotic zone, the amount of living resources such as food decreased, which brings to strong resources competition and environmental stress, and thus the contribution of environmental factors is high for microplanktons. Meanwhile, because of their weak dispersal capability in aphotic water, stochastic processes have a less role in protist distribution.

It is notable that in our VPA, the unexplained proportions of community assembly were high, especially for aphotic community. This means that the ciliate communities are less impacted by spatial and environmental factors but more controlled by other factors. Compared with other habitats like estuary and coast, the open water may provide ciliates with a relatively stable living environment, which thereby reduces the influence of spatial and environmental factors in community assembly. Biological interactions, such as competition, predation and symbiosis played vital roles in ciliate community assembly as revealed by previous studies (Sun *et al.,* 2017; Canals *et al.,* 2020), and might contribute to the unexplained proportion.

### Lower stability of ciliate communities in shelf and aphotic waters

Species interactions are widely regarded as the critical determinants of community distribution patterns and ecosystem functions (Lupatini *et al.,* 2014). Co-occurrence network can reveal the complex interactions among species within microbial communities, are increasingly used to explore and understand the community structuring. Compared with the basin community, the shelf community network has fewer network edges, higher modularity, average path length, and network diameter, lower clustering coefficient, average degree and graph density in our results, indicating a simpler network structure and sparser connections (Yuan *et al.,* 2021).Additionally, in robustness tests, the natural connectivity of the shelf network was lower than basin network during the node removal process. All above results suggested lower structural stability of ciliate network in shelf than basin. Additionally, we found that ciliates in the shelf area exhibited a narrow niche breadth, indicating that the community had lower tolerances for environmental variability and disturbances, which thus also contributed to the community instability. Compared with basin area, shelf area is closer to coast. Disturbed by terrestrial inputs and human activities, the environmental conditions in shelf were less stable, which thus led to the sparse interspecific relationships and low community stability.

Compared to the photic water, the ciliate network in the aphotic water exhibited fewer nodes and edges, higher average path length, network diameter and modularity, but lower average degree and graph density. These indicated relatively sparse connections among species in the aphotic water community. Combined with the lower natural connectivity in robustness tests, our results indicated a lower community stability in the aphotic than photic water. Our findings corroborated those of another study on protistan-bacterial microbiotas in the Western Pacific Ocean where deep water community co-occurrence was less widespread and robust than that in upper water (Sun *et al.,* 2023). Be constrained in the extreme environment, ciliate species diversity is low in the deep water, and thus the interspecific relationships were less built, resulting in a simple community structure. Furthermore, the stable environmental conditions led to the narrow niche breadth of ciliate community as revealed in our results. Low species diversity, simple internal community relationships and narrow niche made the community more fragile when confronted with the environmental changes. Considering the low stability of ciliate community in shelf and aphotic water, our findings suggest that more attention should be paid to these two areas when assessing the influence of climate change and human activities on marine ecosystem.

## CONCLUSIONS

This study examined the spatial distribution, community determinants and species co-occurrence of ciliate communities across the shelf-to-basin continuum in the NESCS. Significant variations in ciliate community composition were found between photic and aphotic waters but not between continental shelf and basin areas. The high nutrients inputs and concentration of food resources in continental shelf and photic waters contributed to higher ciliate abundances than in basin and aphotic waters, respectively. The environmental heterogeneity leads to a more significant driving role of deterministic processes in community assembly across the entire area than stochastic processes. Spatial factors have a more significant impact in community than environmental factors in continental shelf and photic waters, while environmental factors become the main drivers in basin and aphotic waters. Additionally, co-occurrence network analysis indicated that ciliate communities in the continental shelf and aphotic waters exhibited simpler internal community relationships and narrower niche than the basin and photic waters, respectively, which might result in the lower stability of the two former communities. Considering this result, we suggest that more attention should be paid to these two areas when assessing the influence of climate change and human activities on marine ecosystem. Our findings significantly contribute to the understanding of how and why ciliate assembly and co-occurrence change between continental shelf and basin, as well as photic and aphotic waters, offering insight into the dynamics of the microplanktons in the tropical western Pacific oceans.

## Supplementary Material

Supplementary0402_fbaf020

## Data Availability

The data that support the findings of this study are openly available in Science Data Bank at https://doi.org/10.57760/sciencedb.14068

## References

[ref2] Cai, Z., Gan, J., Liu, Z., Hui, C. R. and Li, J. (2020) Progress on the formation dynamics of the layered circulation in the South China Sea. Prog. Oceanogr., 181, 102246. 10.1016/j.pocean.2019.102246.

[ref3] Canals, O., Obiol, A., Muhovic, I., Vaqué, D. and Massana, R. (2020) Ciliate diversity and distribution across horizontal and vertical scales in the open ocean. Mol. Ecol., 29, 2824–2839. 10.1111/mec.15528. 32618376

[ref4] Coppellotti, O. and Matarazzo, P. (2000) Ciliate colonization of artificial substrates in the lagoon of Venice. J. Mar. Biol. Assoc. U. K., 80, 419–427. 10.1017/S0025315400002113.

[ref5] Dai, M., Meng, F., Tang, T., Kao, S., Lin, J., Chen, J., Huang, J., Tian, J. et al. (2009) Excess total organic carbon in the intermediate water of the South China Sea and its export to the North Pacific. Geochem. Geophys. Geosyst., 10, 2009GC002752. 10.1029/2009GC002752.

[ref6] Ding, X., Liu, J., Zhang, H., Ke, Z., Li, J., Liu, W., Li, K., Zhao, C. et al. (2022) Phytoplankton community patterns in the Northeastern South China Sea: implications of intensified Kuroshio intrusion during the 2015/16 El Niño. J. Geophys. Res. Oceans, 127, e2021JC017998. 10.1029/2021JC017998.

[ref7] Dini-Andreote, F., Stegen, J. C., Van Elsas, J. D. and Salles, J. F. (2015) Disentangling mechanisms that mediate the balance between stochastic and deterministic processes in microbial succession. Proc. Natl. Acad. Sci., 112, 1326–1332. 10.1073/pnas.1414261112. PMC4371938 25733885

[ref8] Doherty, M., Tamura, M., Costas, B. A., Ritchie, M. E., McManus, G. B. and Katz, L. A. (2010) Ciliate diversity and distribution across an environmental and depth gradient in Long Island sound, USA. Environ. Microbiol., 12, 886–898. 10.1111/j.1462-2920.2009.02133.x. 20113332

[ref9] Dolan, J. R., Gimenez, A., Cornet-Barthaux, V. and De Verneil, A. (2016) Community structure of Tintinnid ciliates of the microzooplankton in the south West Pacific Ocean: comparison of a high primary productivity with a typical oligotrophic site. J Eukaryotic Microbiology, 63, 813–822. 10.1111/jeu.12328. 27218699

[ref10] Dolan, J. R., Ritchie, M. E. and Ras, J. (2007) The “neutral” community structure of planktonic herbivores, tintinnid ciliates of the microzooplankton, across the SE tropical Pacific Ocean. Biogeosciences, 4, 297–310. 10.5194/bg-4-297-2007.

[ref11] Dufrêne, M. and Legendre, P. (1997) Species assemblages and indicator species:the need for a flexible asymmetrical approach. Ecol. Monogr., 67, 345–366. 10.1890/0012-9615(1997)067[0345.

[ref12] Fernandes, L. F. (2004) Tintininos (Ciliophora, Tintinnina) de águas subtropicais na região Sueste-Sul do Brasil: I. Famílias Codonellidae, Codonellopsidae, Coxliellidae, Cyttarocylidae, Epiplocylidae, Petalotrichidae, Ptychocylidae, Tintinnididae e Undellidae. Revista Brasileira de Zoologia, 21, 551–576. 10.1590/S0101-81752004000300019.

[ref13] Fernandez-Leborans, G. (2001) Relative importance of protozoan functional groups in three marine sublittoral areas. J. Mar. Biol. Assoc. U. K., 81, 735–750. 10.1017/S0025315401004544.

[ref14] Fernandez-Leborans and Fernandez-Fernandez, D. (2002) Protist functional groups in a sublittoral estuarine epibenthic area. Estuaries, 25, 382–392. 10.1007/BF02695981.

[ref15] Gimmler, A., Korn, R., De Vargas, C., Audic, S. and Stoeck, T. (2016) The Tara oceans voyage reveals global diversity and distribution patterns of marine planktonic ciliates. Sci. Rep., 6, 33555. 10.1038/srep33555. 27633177 PMC5025661

[ref16] Gómez, F. (2007) Trends on the distribution of ciliates in the open Pacific Ocean. Acta Oecol., 32, 188–202. 10.1016/j.actao.2007.04.002.

[ref17] Gong, F., Li, G., Wang, Y., Liu, Q., Huang, F., Yin, K. and Gong, J. (2020) Spatial shifts in size structure, phylogenetic diversity, community composition and abundance of small eukaryotic plankton in a coastal upwelling area of the northern South China Sea. J. Plankton Res., 42, 650–667. 10.1093/plankt/fbaa046.

[ref18] Grattepanche, J., Santoferrara, L., McManus, G. and Katz, L. (2015) Distinct assemblage of planktonic ciliates dominates both photic and deep waters on the New England shelf. Mar. Ecol. Prog. Ser., 526, 1–9. 10.3354/meps11256.

[ref19] Grattepanche, J.-D., McManus, G. B. and Katz, L. A. (2016) Patchiness of ciliate communities sampled at varying spatial scales along the New England shelf. PLoS One, 11, e0167659. 10.1371/journal.pone.0167659. 27936137 PMC5147948

[ref20] Herndl, G. J., Brugger, A., Hager, S., Kaiser, E., Obernosterer, I., Reitner, B. and Slezak, D. (1997) Role of ultraviolet-B radiation on bacterioplankton and the availability of dissolved organic matter. In Rozema, J., Gieskes, W. W. C., Van De Geijn, S. C., Nolan, C. and De Boois, H. (eds.), UV-B and Biosphere, Springer, Netherlands, Dordrecht, pp. 42–51.

[ref21] Hu, J., Kawamura, H., Hong, H. and Qi, Y. (2000) A review on the currents in the South China Sea: seasonal circulation, South China Sea warm current and Kuroshio intrusion. J. Oceanogr., 56, 607–624. 10.1023/A:1011117531252.

[ref22] Hu, X., Lin, X. and Song, W. (eds.) (2019) Ciliate Atlas: Species Found in the South China Sea, Springer Singapore, Singapore.

[ref23] Huang, H., Yang, J., Huang, S., Gu, B., Wang, Y., Wang, L., Jiao, N. and Xu, D. (2021) Spatial distribution of planktonic ciliates in the western Pacific Ocean: along the transect from Shenzhen (China) to Pohnpei (Micronesia). Marine Life Science & Technology, 3, 103–115. 10.1007/s42995-020-00075-7. 37073387 PMC10077192

[ref1] Kofoid, C. A. and Campbell, A. S. (1929) A conspectus of the marine and freshwater Ciliata belonging to the suborder Tintinnoinea, with descriptions of new species, principally from the Agassiz expedition to the eastern tropical Pacific, 1904-1905. Univ.Calif.Publ.Zool., 34, 1–403.

[ref25] Kuuppo‑Leinikki, P. and Salonen, K. (1992) Bacterioplankton in a small polyhumic lake with an anoxic hypolimnion. Springer, Dordrecht, 73, pp. 207–219.

[ref26] Li, J., Jiang, X., Li, G., Jing, Z., Zhou, L., Ke, Z. and Tan, Y. (2017) Distribution of picoplankton in the northeastern South China Sea with special reference to the effects of the Kuroshio intrusion and the associated mesoscale eddies. Sci. Total Environ., 589, 1–10. 10.1016/j.scitotenv.2017.02.208. 28273592

[ref27] Li, R., Hu, C., Wang, J., Sun, J., Wang, Y., Jiao, N. and Xu, D. (2021) Biogeographical distribution and community assembly of active Protistan assemblages along an estuary to a basin transect of the northern South China Sea. Microorganisms, 9, 351. 10.3390/microorganisms9020351. 33578968 PMC7916720

[ref28] Liu, W., Cheng, Z., Wen, S., Li, G., Ke, Z., Li, D. and Tan, Y. (2024) Effects of temporal, spatial, and environmental factors on ciliates community in northeastern South China Sea, with notes on co-occurrence patterns of environment, phytoplankton, and ciliate. Microbiol Spectr, 13, e01247-24. 10.1128/spectrum.01247-24. PMC11705822 39611825

[ref29] Liu, W., McManus, G. B., Lin, X., Huang, H., Zhang, W. and Tan, Y. (2021) Distribution patterns of ciliate diversity in the South China Sea. Front. Microbiol., 12, 689688. 10.3389/fmicb.2021.689688. 34539599 PMC8446678

[ref30] Lupatini, M., Suleiman, A. K. A., Jacques, R. J. S., Antoniolli, Z. I., De Siqueira Ferreira, A., Kuramae, E. E. and Roesch, L. F. W. (2014) Network topology reveals high connectance levels and few key microbial genera within soils. Front. Environ. Sci., 2, 10. 10.3389/fenvs.2014.00010.

[ref31] Lynn, D. H. (2010) The Ciliated Protozoa. Springer, Netherlands, Dordrecht.

[ref32] Mellard, J. P., Yoshiyama, K., Klausmeier, C. A. and Litchman, E. (2012) Experimental test of phytoplankton competition for nutrients and light in poorly mixed water columns. Ecol. Monogr., 82, 239–256. 10.1890/11-0273.1.

[ref33] Wilbert, N. (1975) Eine verbesserte technik der protargolimpra gnation fur ciliaten. Mikrokosmos, 64, 171–179. 10.3389/fmicb.2021.689688.

[ref34] Nan, F., Xue, H. and Yu, F. (2015) Kuroshio intrusion into the South China Sea: a review. Prog. Oceanogr., 137, 314–333. 10.1016/j.pocean.2014.05.012.

[ref35] Niu, K., Liu, N., Shen, Z., He, F. and Fang, J. (2009) Neutral and niche theories of community assembly. Biodivers. Sci., 17, 579–593 (in Chinese).

[ref36] Pandit, S. N., Kolasa, J. and Cottenie, K. (2009) Contrasts between habitat generalists and specialists: an empirical extension to the basic Metacommunity framework. Ecology, 90, 2253–2262. 10.1890/08-0851.1. 19739387

[ref37] Peng, G. and Wu, J. (2016) Optimal network topology for structural robustness based on natural connectivity. Physica A, 443, 212–220. 10.1016/j.physa.2015.09.023.

[ref38] Pitta, P., Giannakourou, A. and Christaki, U. (2001) Planktonic ciliates in the oligotrophic Mediterranean Sea: longitudinal trends of standing stocks, distributions and analysis of food vacuole contents. Aquat. Microb. Ecol., 24, 297–311. 10.3354/ame024297.

[ref39] Pomeroy, L. R. (1974) The Ocean’s food web, a changing paradigm. Bioscience, 24, 499–504. 10.2307/1296885.

[ref40] Pratt, J. R. and Cairns, J. (1985) Functional groups in the protozoa: roles in differing ecosystems. J. Protozool., 32, 415–423. 10.1111/j.1550-7408.1985.tb04037.x.

[ref40a] R Core Team (2021). R: A language and environment for statistical computing. R Foundation for Statistical Computing, Vienna, Austria.

[ref41] Santoferrara, L. and Alder, V. (2009) Abundance trends and ecology of planktonic ciliates of the south-western Atlantic (35-63 S): a comparison between neritic and oceanic environments. J. Plankton Res., 31, 837–851. 10.1093/plankt/fbp033.

[ref42] Santoferrara, L. F., Grattepanche, J.-D., Katz, L. A. and McManus, G. B. (2016) Patterns and processes in microbial biogeography: do molecules and morphologies give the same answers? The ISME Journal, 10, 1779–1790. 10.1038/ismej.2015.224. 26849313 PMC4918432

[ref43] Santoferrara, L. F., Rubin, E. and Mcmanus, G. B. (2018) Global and local DNA (meta)barcoding reveal new biogeography patterns in tintinnid ciliates. J. Plankton Res., 40, 209–221. 10.1093/plankt/fby011.

[ref44] Sherr, E. B. and Sherr, B. F. (2002) Significance of predation by protists in aquatic microbial food webs. Antonie Van Leeuwenhoek, 81, 293–308. 12448728 10.1023/a:1020591307260

[ref44a] Su, J . (2004) Overview of the South China Sea circulation and its influence on the coastal physical oceanography outside the Pearl River Estuary. Cont. Shelf Res., 24, 1745–1760. 10.1016/j.csr.2004.06.005.

[ref45] Sun, P., Huang, L., Xu, D., Huang, B., Chen, N. and Warren, A. (2017) Marked seasonality and high spatial variation in estuarine ciliates are driven by exchanges between the ‘abundant’ and ‘intermediate’ biospheres. Sci. Rep., 7, 9494. 10.1038/s41598-017-10308-y. 28842665 PMC5573402

[ref46] Sun, P., Huang, X., Wang, Y. and Huang, B. (2021) Protistan-bacterial microbiota exhibit stronger species sorting and greater network connectivity offshore than nearshore across a coast-To-Basin continuum. mSystems, 6, e00100–e00121. 10.1128/msystems.00100-21. 34636671 PMC8510552

[ref47] Sun, P., Wang, Y., Laws, E. and Huang, B. (2020) Water mass–driven spatial effects and environmental heterogeneity shape microeukaryote biogeography in a subtropical, hydrographically complex ocean system - a case study of ciliates. Sci. Total Environ., 706, 135753. 10.1016/j.scitotenv.2019.135753. 31836222

[ref48] Sun, P., Wang, Y., Zhang, Y., Logares, R., Cheng, P., Xu, D. and Huang, B. (2023) From the sunlit to the aphotic zone: assembly mechanisms and Co-occurrence patterns of Protistan-bacterial microbiotas in the western Pacific Ocean. mSystems, 8, e00013–e00023. 10.1128/msystems.00013-23. 36847533 PMC10134807

[ref49] Suzuki, T. and Taniguchi, A. (1995) Sinking rate of loricae of some common tintinnid ciliates. Fish. Oceanogr., 4, 257–263. 10.1111/j.1365-2419.1995.tb00149.x.

[ref50] Telesford, Q. K., Joyce, K. E., Hayasaka, S., Burdette, J. H. and Laurienti, P. J. (2011) The ubiquity of small-world networks. Brain Connect., 1, 367–375. 10.1089/brain.2011.0038. 22432451 PMC3604768

[ref51] Wang, C., Zhao, C., Zhou, B., Xu, Z., Ma, J., Li, H., Wang, W., Chen, X. et al. (2024) Latitudinal pronounced variations in tintinnid (Ciliophora) community at surface waters from the South China Sea to the Yellow Sea: oceanic-to-neritic species shift, biotic-abiotic interaction and future prediction. Sci. Total Environ., 912, 169354. 10.1016/j.scitotenv.2023.169354. 38104840

[ref52] Wu, W., Lu, H.-P., Sastri, A., Yeh, Y.-C., Gong, G.-C., Chou, W.-C. and Hsieh, C.-H. (2018) Contrasting the relative importance of species sorting and dispersal limitation in shaping marine bacterial versus protist communities. The ISME Journal, 12, 485–494. 10.1038/ismej.2017.183. 29125596 PMC5776463

[ref53] Xu, D., Kong, H., Yang, E.-J., Wang, Y., Li, X., Sun, P., Jiao, N., Lee, Y. et al. (2022) Spatial dynamics of active microeukaryotes along a latitudinal gradient: diversity, assembly process, and co-occurrence relationships. Environ. Res., 212, 113234. 10.1016/j.envres.2022.113234. 35390306

[ref54] Xu, H., Min, G.-S., Choi, J.-K., Jung, J.-H. and Park, M.-H. (2009) An approach to analyses of periphytic ciliate colonization for monitoring water quality using a modified artificial substrate in Korean coastal waters. Mar. Pollut. Bull., 58, 1278–1285. 10.1016/j.marpolbul.2009.05.003. 19497590

[ref55] Xue, H., Chai, F., Pettigrew, N., Xu, D., Shi, M. and Xu, J. (2004) Kuroshio intrusion and the circulation in the South China Sea. J. Geophys. Res., 109, 2002JC001724. 10.1029/2002JC001724.

[ref56] Yang, J., Huang, S., Fan, W., Warren, A., Jiao, N. and Xu, D. (2020) Spatial distribution patterns of planktonic ciliate communities in the East China Sea: potential indicators of water masses. Mar. Pollut. Bull., 156, 111253. 10.1016/j.marpolbul.2020.111253. 32510395

[ref57] Yang, S., Yang, Q., Li, X., Chao, X., Liu, H., Wei, L. and Ba, S. (2023) Deterministic processes dominate the geographical distribution and community assembly of phytoplankton in typical plateau rivers. Biodivers. Sci., 31, 43–57 (in Chinese).

[ref58] Yu, X., Li, X., Liu, Q., Yang, M., Wang, X., Guan, Z., Yang, J., Liu, M. et al. (2022) Community assembly and co-occurrence network complexity of pelagic ciliates in response to environmental heterogeneity affected by sea ice melting in the Ross Sea. Antarctica. Science of The Total Environment, 836, 155695. 10.1016/j.scitotenv.2022.155695. 35525347

[ref59] Yuan, M. M., Guo, X., Wu, L., Zhang, Y., Xiao, N., Ning, D., Shi, Z., Zhou, X. et al. (2021) Climate warming enhances microbial network complexity and stability. Nat. Clim. Chang., 11, 343–348. 10.1038/s41558-021-00989-9.

[ref60] Zhang, C., Sun, J., Wang, D., Song, S., Zhang, X. and Munir, S. (2017) Tintinnid community structure in the eastern equatorial Indian Ocean during the spring inter-monsoon period. Aquat. Biol., 26, 87–100. 10.3354/ab00677.

[ref61] Zhao, R., Zhao, F., Feng, L., Fang, J. K., Liu, C. and Xu, K. (2023) A deep seamount effect enhanced the vertical connectivity of the planktonic community across 1,000 m above summit. *JGR*. Oceans, 128, e2022JC018898.

[ref62] Zhou, J. and Ning, D. (2017) Stochastic community assembly: does it matter in microbial ecology? Microbiol. Mol. Biol. Rev., 81, mmbr.00002-17. 10.1128/MMBR.00002-17. PMC5706748 29021219

[ref63] Zhu, C., Liu, W., Li, X., Xu, Y., El-Serehy, H. A., Al-Farraj, S. A., Ma, H., Stoeck, T. et al. (2021) High salinity gradients and intermediate spatial scales shaped similar biogeographical and co-occurrence patterns of microeukaryotes in a tropical freshwater-saltwater ecosystem. Environ. Microbiol., 23, 4778–4796. 10.1111/1462-2920.15668. 34258839

