# Supplementary material for: Distribution patterns and driving mechanisms of ciliate communities from continental shelf to deep basin of the Northeastern South China Sea
Source: J Plankton Res. 2025 Aug 12;47(5):fbaf020. doi: 10.1093/plankt/fbaf020 (PMC12343036; doi:10.1093/plankt/fbaf020)
Supplement: Supplementary0402_fbaf020 [file supplementary0402_fbaf020.docx]

**Supplementary Data**

**Figure S1-S7**


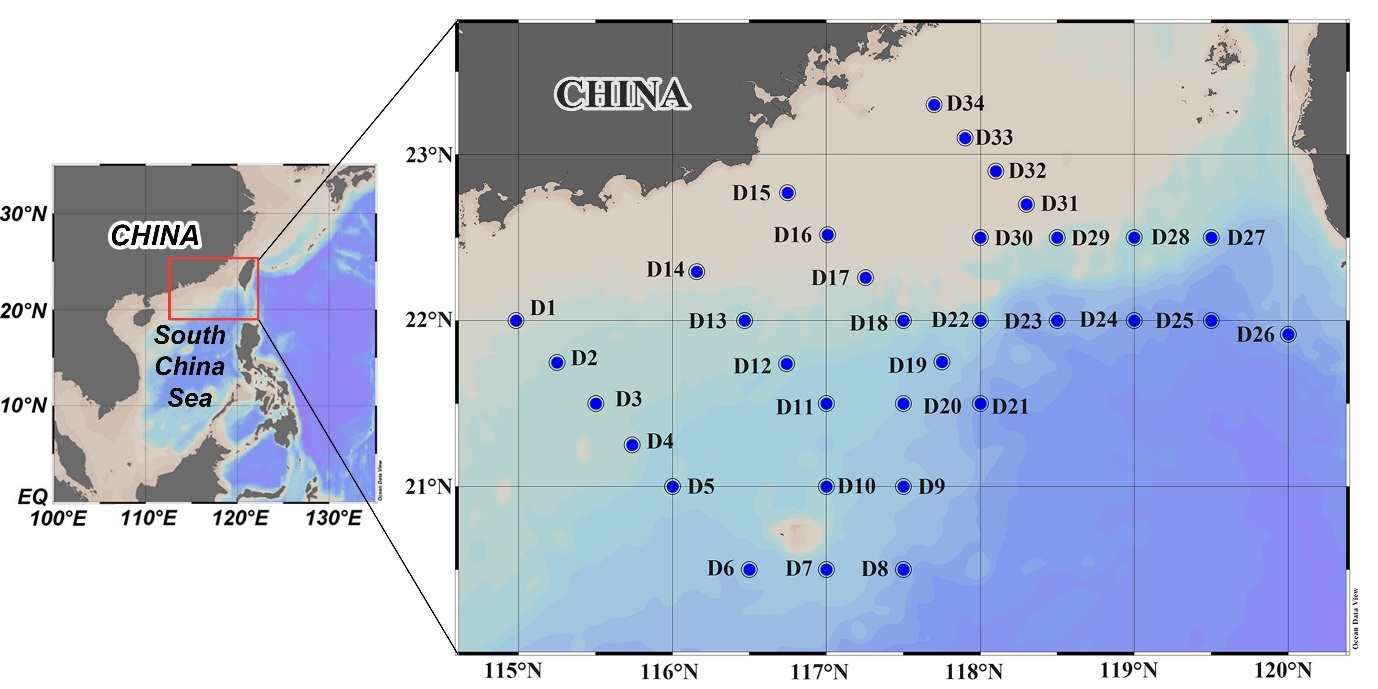


Fig. S1. Sampling stations in the northeastern South China Sea during the winter of 2013.


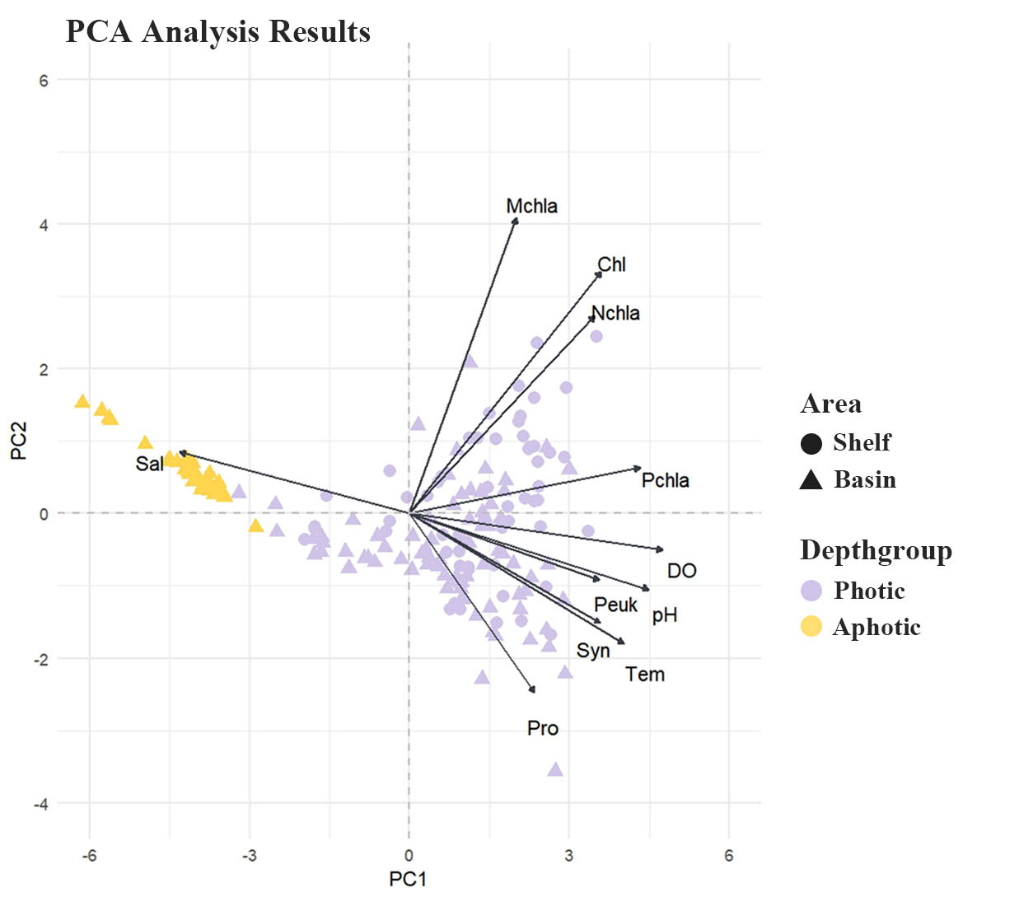


Fig. S2. Principal Component Analysis (PCA) results of environmental data.


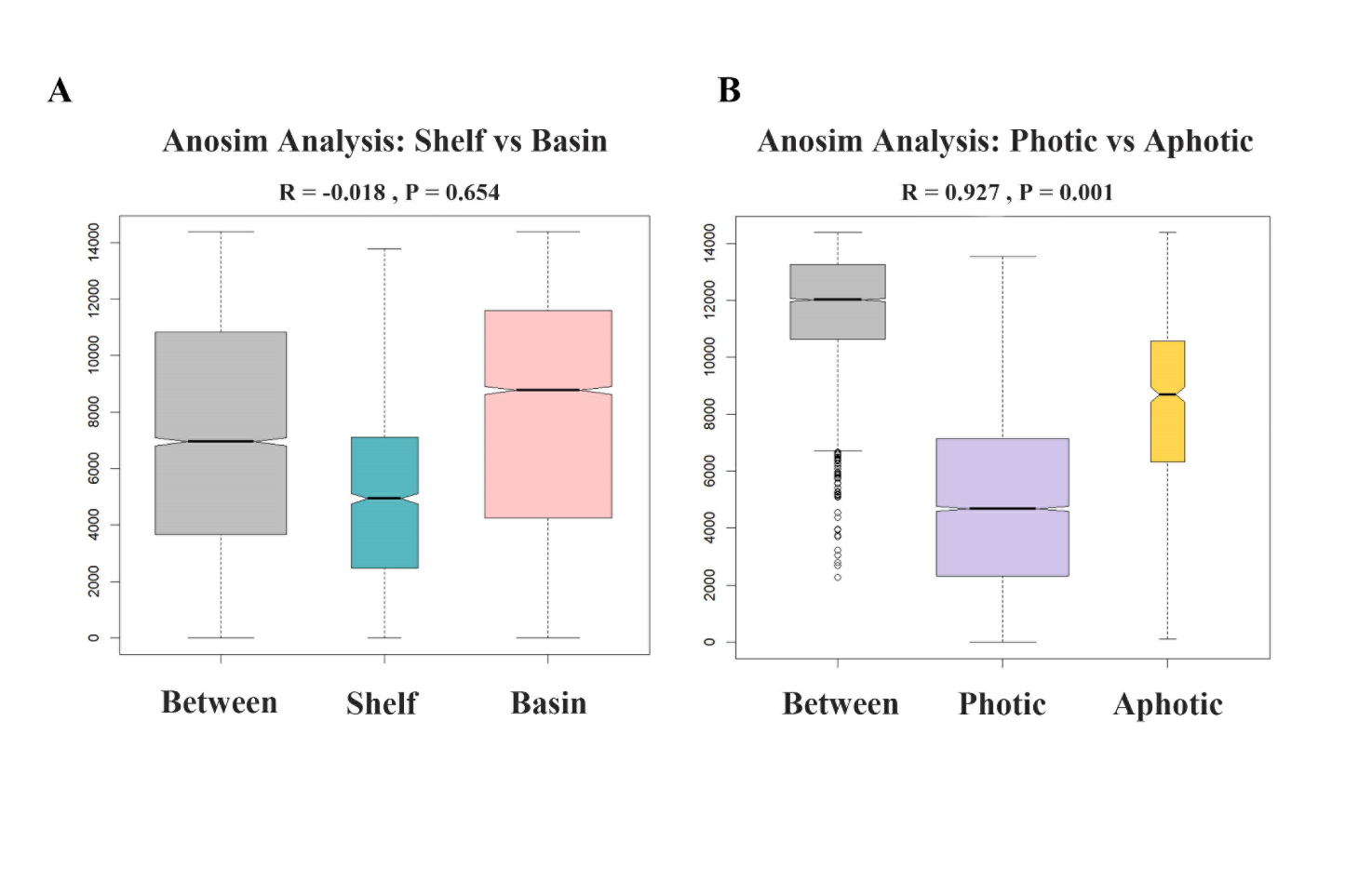


Fig. S3. ANOSIM analysis results of ciliate community grouping in continental shelf and basin areas (A) and in photic and aphotic zones (B).


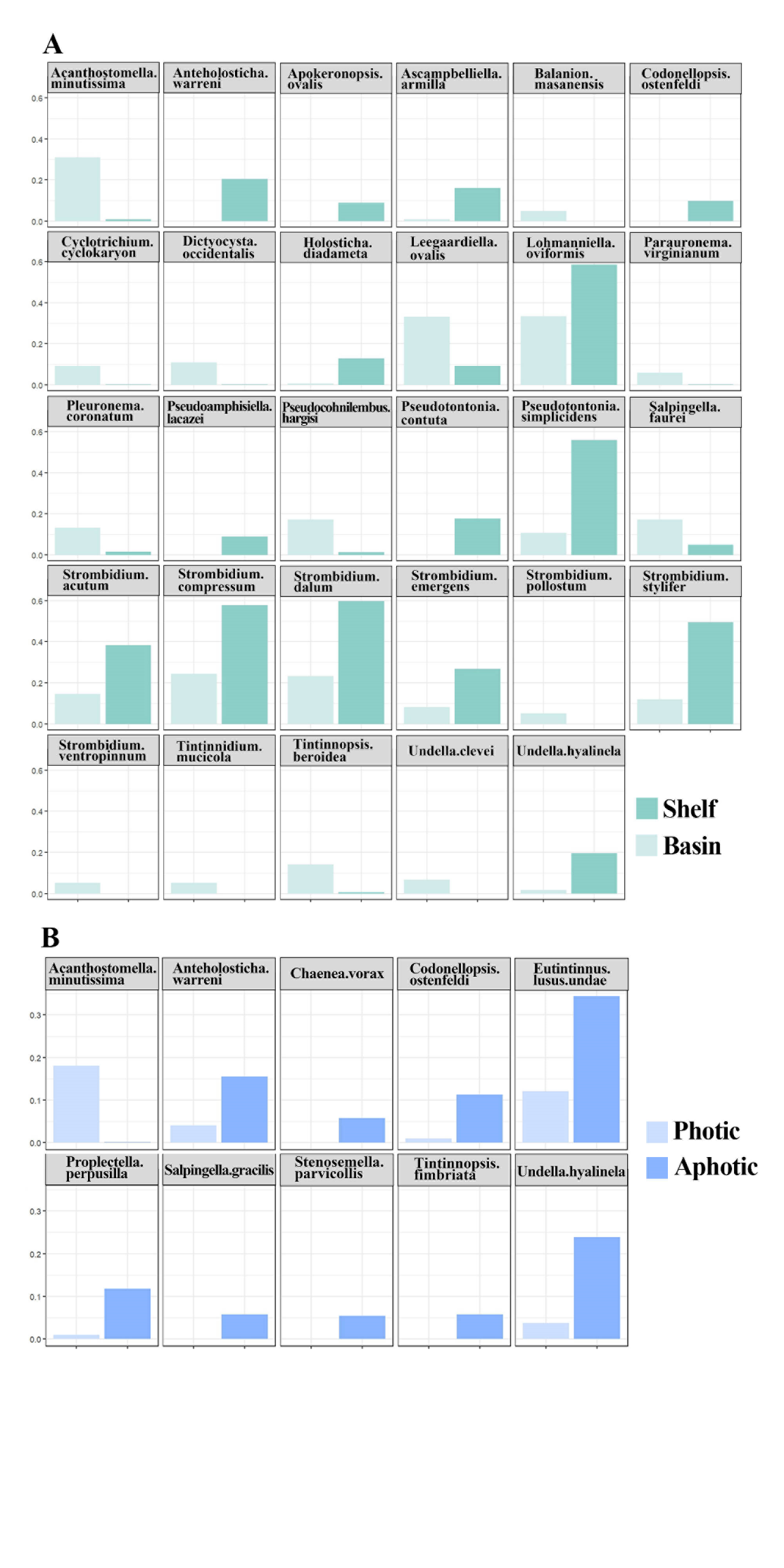


Fig. S4. Indicator species of ciliate communities in continental shelf and basin areas (A), and in photic and aphotic zones (B).


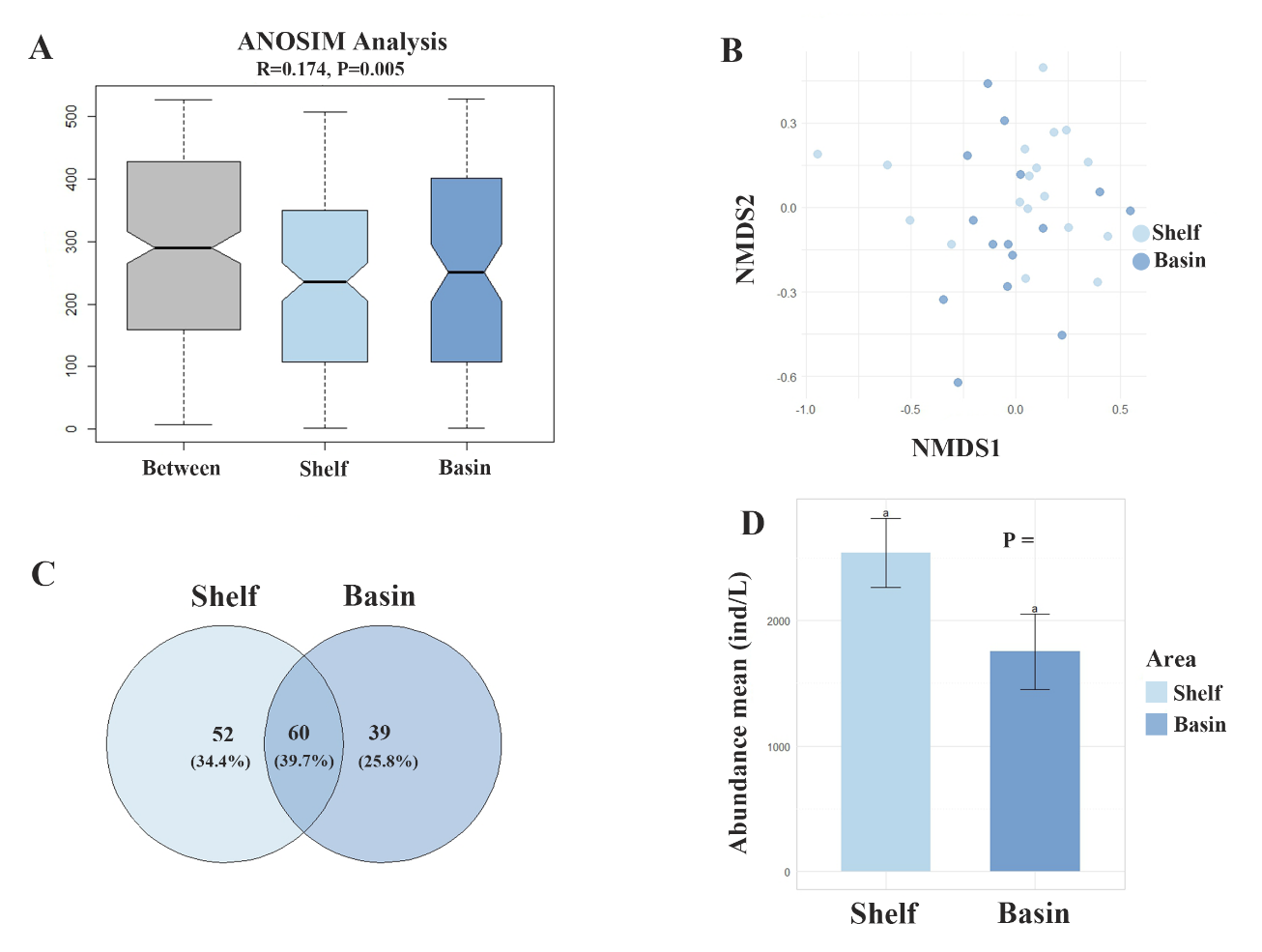


Fig. S5. Comparison of ciliate communities in different spatial groups in the northeastern South China Sea based on surface data from the upper 200 m. Anosim and NMDS analysis showing the distribution of ciliate samples in Shelf and Basin areas (A) (B); Proportions of shared and endemic ciliate species in Shelf and Basin areas (C); Comparison of ciliate abundance in Shelf and Basin areas (D).


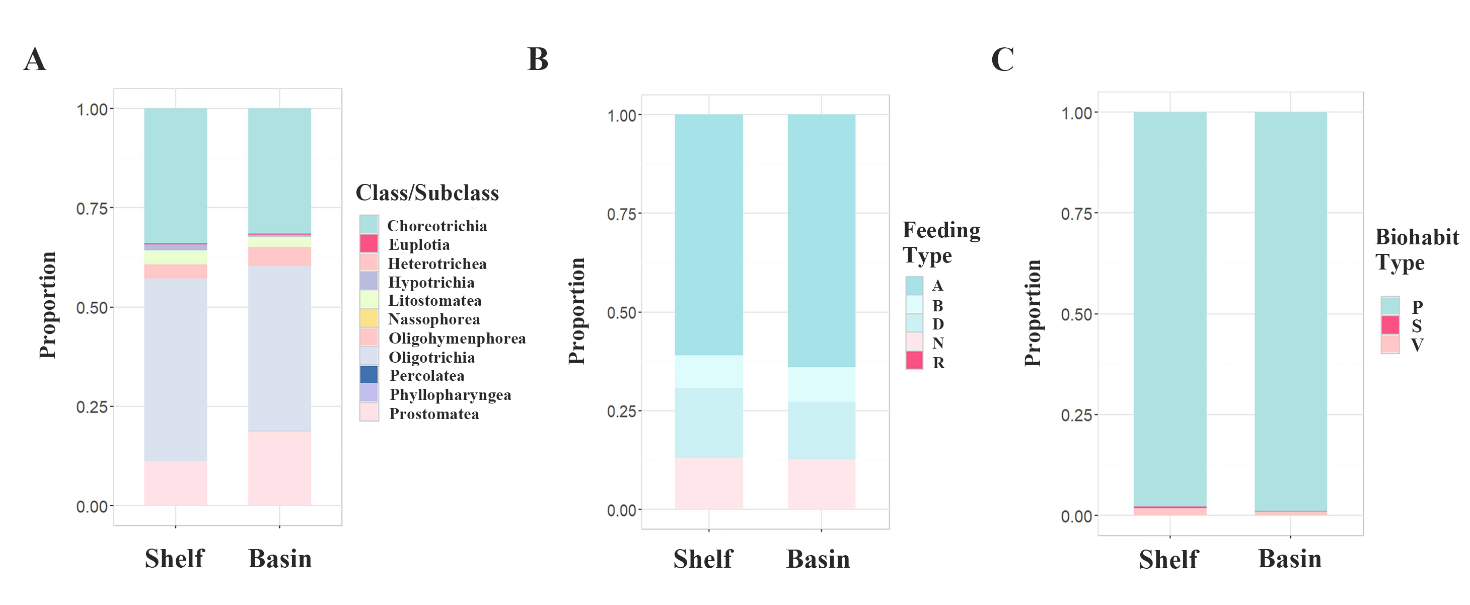


Fig. S6. Comparison of ciliate species composition in different spatial groups in the northeastern South China Sea based on surface data from the upper 200 m. Taxonomic composition at class/subclass level to show their relative abundance in Shelf and Basin areas (A); Feeding type composition in Shelf and Basin areas (B); Biohabit type compositions in Shelf and Basin areas (C). A (Algivore), B (Bacterivore), D (Detritivore), N (Non-selective), R (Raptor), P (Planktonic), S (Sessile), and V (Vagile).


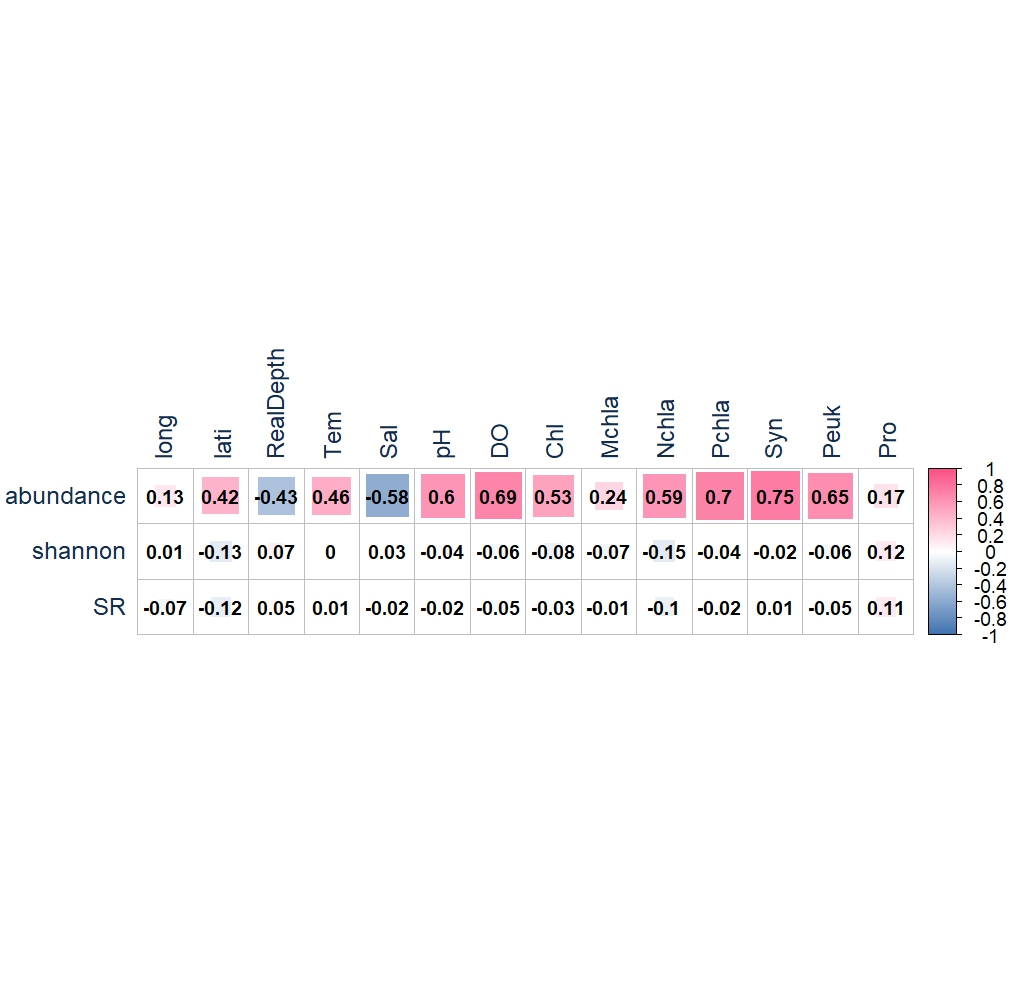


Fig. S7. Pearson correlation analysis results between environmental factors and ciliate abundance, Shannon index, species richness (SR) across the entire sea area.
